# Speckle tracking ultrasound as a new tool to predict the weaning outcome of mechanical ventilation patients: a prospective observational study

**DOI:** 10.3389/fmed.2024.1449938

**Published:** 2024-12-06

**Authors:** Rui Li, Yao Zhou, Wan Chen, Liwen Lyu, Guozheng Qiu, Chunxi Pan, Yutao Tang

**Affiliations:** Department of Emergency, Guangxi Academy of Medical Sciences & People’s Hospital of Guangxi Zhuang Autonomous Region, Nanning, China

**Keywords:** mechanical ventilation, diaphragmatic function, speckle tracking ultrasonography, outcome, weaning

## Abstract

**Introduction:**

Speckle tracking ultrasound is a novel technique for evaluating diaphragm movement, yet its guidance in weaning mechanically ventilated patients remains unclear. In this study, we assessed diaphragmatic function using speckle tracking ultrasound and guided the weaning process.

**Methods:**

A total of 86 mechanically ventilated patients were included and divided into successful or failed weaning groups. Diaphragmatic function was assessed using speckle tracking ultrasound, M-ultrasound diaphragm excursion (DE), and diaphragmatic twitch force (DTF) after 30 min spontaneous breathing trial (SBT). The diagnostic performance of these indicator in predicting weaning outcomes was also evaluated.

**Results:**

In this study, a total of 86 patients completed the follow-up for weaning outcomes, with 35 cases of weaning failure and 51 cases of successful weaning. Logistic regression analysis identified whole strain (*p* = 0.037) and DE (*p* = 0.004) as independent predictors of weaning outcome. Receiver operating characteristic (ROC) curve showed that the strain threshold for Costal Diaphragm (Dlcos) was −9.836, Area Under the Curve (AUC) value was 0.760, the predictive specificity for weaning failure was 72.5%, and the sensitivity was 80%. DE value exceeding 1.015 cm had an AUC value of 0.785, noting that DE value had a high specificity (90.2%) for predicting successful weaning, but a lower sensitivity (60%). After merging, the AUC of whole strain and DE was 0.856, and the sensitivity (80%) and specificity (80.4%) were more balanced compared to using DE alone.

**Conclusion:**

The findings of this study demonstrate the feasibility of using speckle tracking ultrasound to assess diaphragmatic function in mechanically ventilated patients. The combined utilization of whole strain and DE provides a more precise evaluation of diaphragmatic function in ICU patient, which may improve patient outcome.

## Introduction

Identifying the optimal timing for weaning mechanically ventilated patients remains a clinical challenge. Approximately 30% of patients experience premature or delayed weaning, leading to an increased risk of ventilator-related complications such as pneumonia, tracheal injury, and barotrauma (1). Current guidelines recommend a spontaneous breathing trial (SBT) as a predictive tool for weaning outcomes (1). However, even if patients pass the SBT and are successfully extubated, a significant proportion (15 to 20%) may still require re-intubation (2, 3). What’s more, pulmonary edema, resulting in SBT, can lead to a reduction in effective pulmonary ventilation and changes in lung compliance, thereby exacerbating post-extubation respiratory distress and diaphragmatic dysfunction (4, 5). It is worth noting that currently there is no singular reliable marker to accurately predict weaning outcomes.

In recent years, ultrasound has emerged as a non-invasive and effective technique for assessing pulmonary ventilation and diaphragmatic function, widely applied in critically ill and perioperative patients (6–8). In clinical practice, M-mode ultrasound is commonly utilized to measure diaphragm displacement and thickening fraction for evaluating diaphragm functionality and guiding weaning decisions (9). Speckle tracking ultrasound, as an emerging technology, has been validated for evaluating diaphragm functionality in healthy volunteers (10, 11), showing good correlations with gold-standard measures such as transdiaphragmatic pressure and electromyography (12). However, there is a paucity of literature discussing the predictive role of speckle tracking ultrasound in weaning outcomes among critically ill patients.

This study aims to investigate the potential and clinical significance of Speckle tracking ultrasound in evaluating diaphragmatic function and predicting weaning outcomes among mechanically ventilated patients in critical care unit (ICU). We hypothesize that Speckle tracking ultrasound offers a more precise and comprehensive assessment of diaphragm function compared to traditional methods, ultimately leading to improved weaning predictions.

## Materials and methods

### Subjects

This prospective, single-center observational study was conducted in the Emergency Intensive Care Unit (EICU) of the People’s Hospital of Guangxi Zhuang Autonomous Region. The study protocol was approved by the Ethics Committee of the People’s Hospital of Guangxi Zhuang Autonomous Region (KY-GZR-2022-047). Written informed consent was obtained from all participants’ family members. Inclusion criteria were as follows: aged ≥18 years, received mechanical ventilation (MV) for more than 48 h, and deemed suitable for SBT. Exclusion criteria were as follows: patients with pre-existing neuromuscular disorders, diaphragmatic paralysis, cervical injuries, pneumothorax, history of major cardiac, thoracic, and abdominal surgery, mediastinal emphysema, and those with poor echogenicity or who were unable to tolerate ultrasound examination.

### Ultrasound imaging and analysis

**Figure 1:**
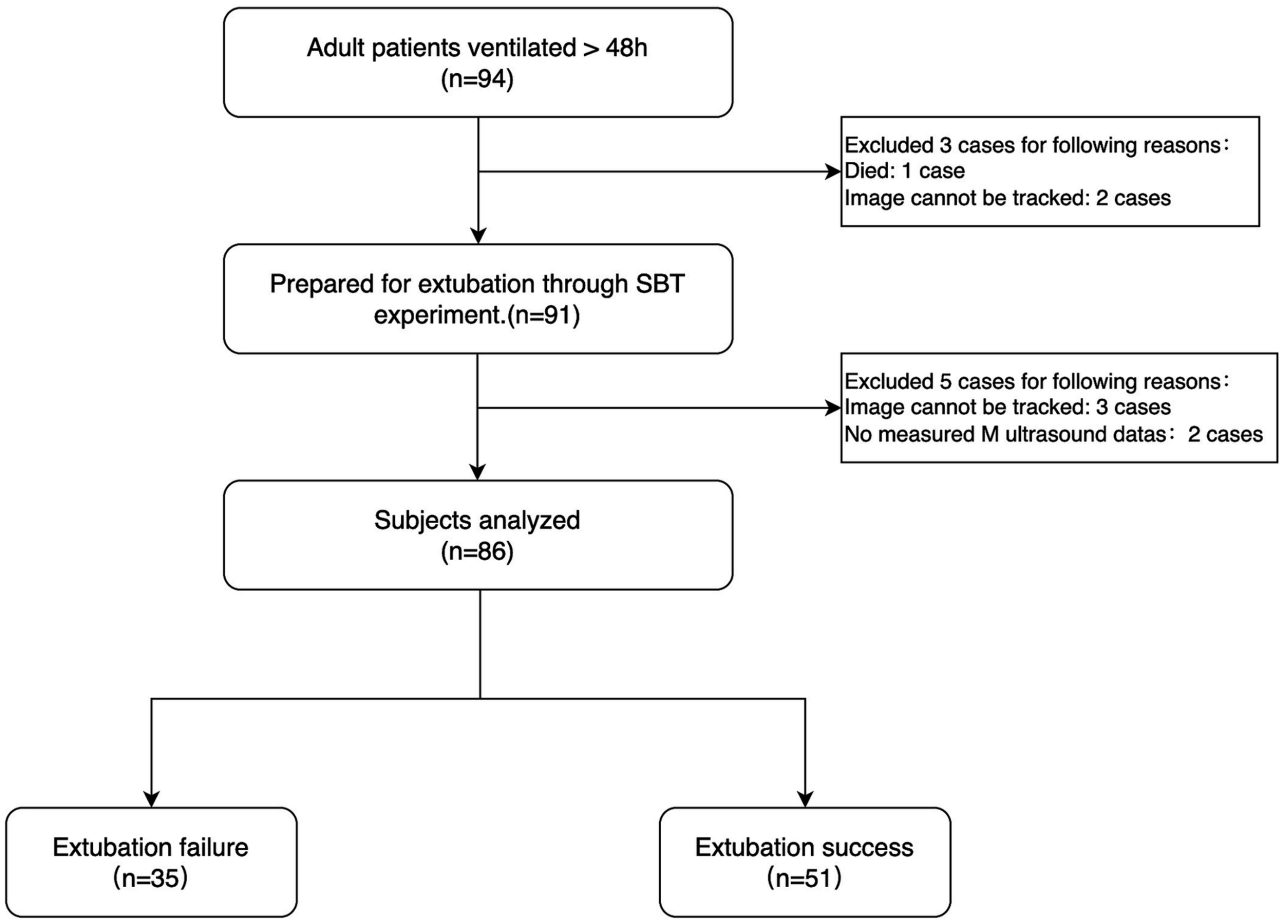
Flowchart of this study.

All enrolled patients underwent SBT under low-level pressure support ventilation (PSV) before weaning. The specific parameters were as follows: positive end-expiratory pressure (PEEP) < 8 cm H2O, pressure support (PS) ranging from 5 to 8 cm H2O, and FiO2 at 35% (13). After successful completion of SBT, patients remained in a supine position with the head of the bed elevated at a 30° angle. The diaphragmatic strain was measured using speckle tracking ultrasound by YT, a senior physician with specialized ultrasound training. DE and DTF were measured using M-mode ultrasonography. If SBT failed, mechanical ventilation was continued, and a comprehensive cardiopulmonary ultrasound examination (CCUE) protocol (14) was performed to assess cardiac and pulmonary function, aiming to exclude underlying cardiopulmonary dysfunction as the cause of weaning failure, The intensivist specialized doctor was blinded to the Diaphragm measurement results of the patient. Detailed experimental procedures are shown in Figure 1.

This study adapted the M9 color Doppler ultrasound diagnostic system (Mindray Biomedical Co., Ltd., Shenzhen, China). During the procedure, the ultrasound machine is adjusted to ensure an appropriate gain and depth (where the gain is optimized to visualize the aperture of the highlight echo and the depth is set to approximately 10–15 cm). The frame rate is maintained at 50 frames per second, ensuring smooth imaging. ECG recordings are synchronized with the ultrasound at a rate of 50 millimeters per second. In B-mode, the ultrasound probe is positioned near the junction of the right midclavicular line and costal margin, allowing for simultaneous visualization of the liver, inferior vena cava, and the hyperechoic diaphragm. The bright echogenic layer adjacent to the liver represents the peritoneum, while the bright echogenic layer close to the lungs corresponds to the pleura. The diaphragm lies between these two structures. All measurements are taken when the patient’s breathing is relatively stable and the ultrasound image is consistent.

### Ultrasonographic assessment of diaphragm excursion

The patient was positioned in the supine posture. The ultrasound probe (SP5-1 s array probe, Mindray Biomedical Co., Ltd., Shenzhen, China) was positioned adjacent to the right midclavicular line and the right costal margin, with the ultrasound window focused on the liver. The ultrasound beam was aligned perpendicular to the posterior third of the diaphragm, revealing a dense echogenic shadow at the lung-liver junction.

### Acquisition of diaphragm thickening fraction

The diaphragm thickness, defined as the distance between the pleura and peritoneum at the thoracic junction, undergoes changes during respiration. To quantify this, the rate of change in diaphragm thickness is employed, reflecting the respiratory effort and efficiency of diaphragm contraction. For measurement, a SP5-1 s probe is selected and placed in the 8th to 10th intercostal space, intersecting the midaxillary line or anterior axillary line. Here, the probe is positioned perpendicular to the chest wall, revealing two parallel echogenic layers: the pleural layer proximal to the skin and the peritoneal layer slightly deeper. The lower echo area between these layers represents the diaphragm. Using these measurements, the rate of diaphragm thickness change is calculated as DTF = [end-inspirational diaphragm thickness (DTei) − end-expiratory diaphragm thickness (DTee)] / DTei × 100%. This metric offers valuable insights into respiratory mechanics and diaphragm function.

### Speckle tracking imaging

**Figure 2:**
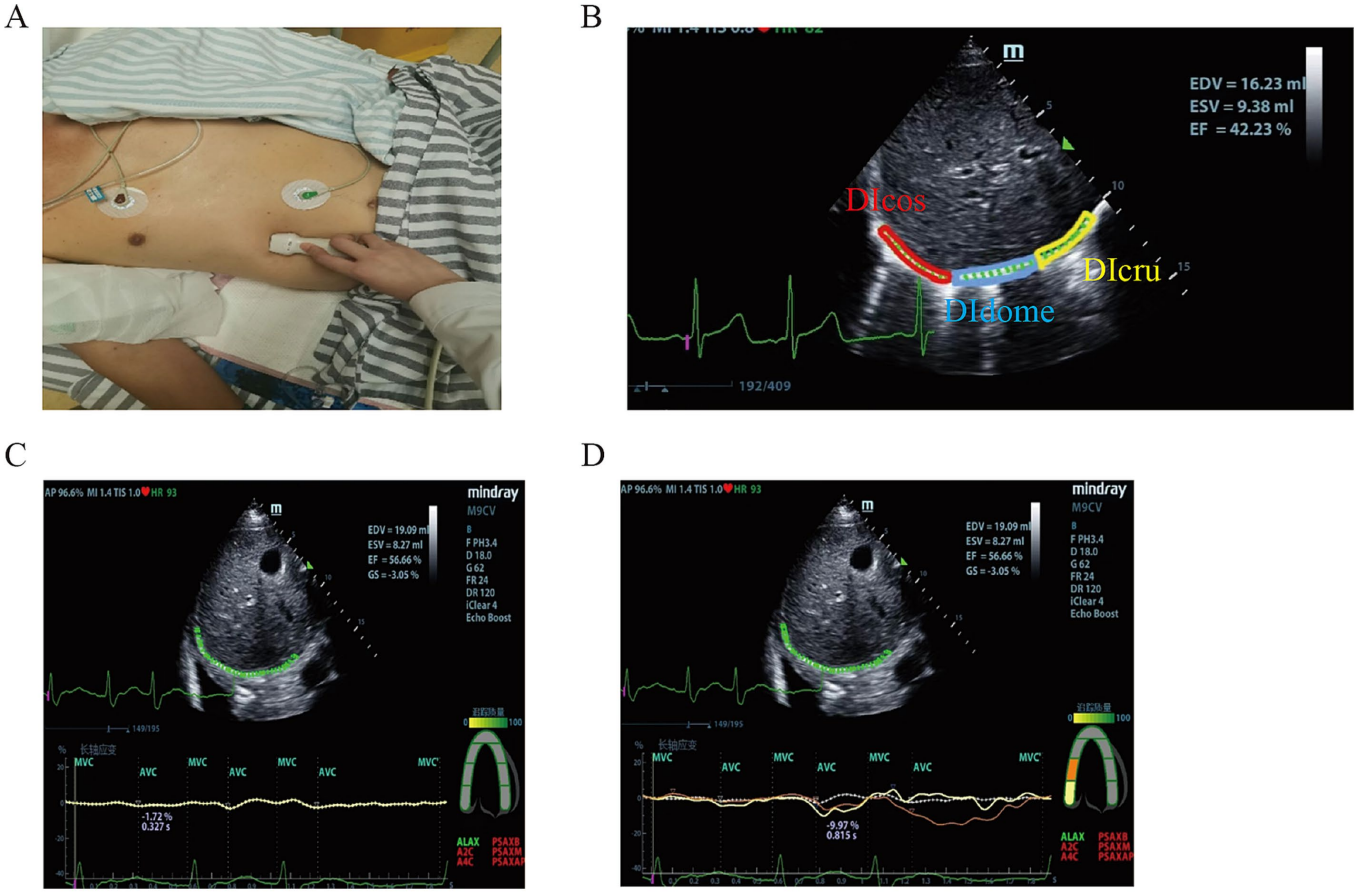
Speckle tracking imaging. **(A)** Speckle tracking location diagram; **(B)** Segmentation of diaphragm by speckle tracking; **(C)** Speckle tracking whole strain parameters; **(D)** Speckle tracking parameters.

B-mode ultrasound was used to obtain diaphragmatic images. The right hepatic vein and liver aided in identifying the anatomical structure of the diaphragm. A Mindy M9 color Doppler ultrasound diagnostic device (C5-1s array probe, Mindray Biomedical Co., Ltd., Shenzhen, China) was used to record clips covering three respiratory cycles, at least 12 s. The built-in speckle tracking software of the ultrasound machine was then employed to select a stable inspiratory phase and manually delineate three regions of interest (ROI): Costal Diaphragm (Dlcos), diaphragmatic dome (DIdome) and Crural Diaphragm (DIcru). At least seven points were manually marked within each ROI. By double-clicking the mouse, the tracking points were automatically generated for the ROI. Appropriate thickness was selected, and manual adjustments were made to align the edges and midline of the ROI with the boundaries of the pleura, diaphragm, and peritoneum. This process was repeated for all three ROIs to ensure accurate point tracking analysis. The longitudinal strain analysis model along the parasternal long axis was chosen for analysis, with segmental adjustments made to match the diaphragmatic segments under the long-axis pattern. Tracking was initiated by clicking the “Start Tracking” button, ensuring that at least 80% of the diaphragmatic speckles were trackable. Re-drawing or re-tracking was allowed in this study, and images with incomplete tracking were discarded. The operational procedure is illustrated in Figure 2.

### Outcomes

The weaning outcome was determined to be successful if the patient maintained spontaneous breathing for ≥48 h without the need for any level of ventilator support following the first time after SBT extubation. Otherwise, the outcome was classified as a weaning failure. Patients were categorized into two groups: weaning failure and weaning success. Factors known to influence weaning outcomes, including underlying diseases, duration of ventilation, weaning time, and relevant blood biochemistry findings, were recorded.

### Statistical analysis

SPSS version 26 (IBM Corp., Armonk, NY, United States) for statistical analysis. The K-S test analyses whether the measurement data conformed to the normal distribution. The measurement data to the normal distribution were expressed as the mean ± standard deviation, and the count data were expressed as the percentage (%). the comparison between groups was performed using the χ^2^ test; the quantitative data that did not conform to the normal distribution was represented by M (P25, P75), and the Mann–Whitney U test was used between the two groups. This study employed a stepwise regression approach to select variables that independently influence the outcome, and subsequently constructed a multivariate logistic regression model with the variables ultimately identified. The receiver operating characteristic curve (ROC) was drawn to evaluate the indicators for predicting the outcome of weaning, and *p* < 0.05 was considered statistically significant. The correlations between speckle tracking ultrasound measurements and M mode ultrasound were calculated using the Spearman method. Two-tailed *p*-values less than 0.05 were considered to indicate statistical significance.

## Results

This study enrolled a total of 86 patients, with an average age of 59.55 ± 13.61 years, including 32 females (30.72%). The main causes for mechanical ventilation were acute cerebrovascular disease (*n* = 20, 23.3%), acute heart failure (*n* = 23, 26.7%), postoperative (*n* = 10, 11.6%), and severe pneumonia (*n* = 24, 27%). The 86 patients were divided into two groups: a successful weaning group with 51 cases and a weaning failure group with 35 cases. Except for the duration of mechanical ventilation, there were no significant differences in the baseline data between the two groups. Table 1 shows the baseline characteristics of patients classified by weaning outcomes.

**Table 1:** The baseline characteristics of patients.

Characteristic	Weaning success group (*n* = 51)	Weaning failure group (*n* = 35)	*t/χ^2^/Z*-Values	*p*-Values
Male sex (*n*, %)	31 (60.78)	23 (65.71)	*0.216*	0.642
Age (year) (Y, *Mean ± SD*)	57.62 ± 14	62.37 ± 12	−1.602	0.113
BMI (kg/m^2^, *Mean ± SD*)	22.33 ± 4	22.63 ± 3	−0.406	0.686
APACHEII (*Mean ± SD*)	21.63 ± 10	24.54 ± 10	−1.375	0.173
SOFA (*Mean ± SD*)	7.73 ± 4	7.91 ± 3	−0.242	0.809
HR (*Mean ± SD*)	91.76 ± 18	89.91 ± 14	0.519	0.605
MAP (mmHg, *Mean ± SD*)	89.51 ± 13	85.45 ± 10	*1.635*	0.106
RR (*Mean ± SD*)	22 ± 1.98	23 ± 2.51	−1.184	0.240
VT (L, *Mean ± SD*)	0.489 ± 0.11	0.471 ± 0.13	0.626	0.533
EF (%)	54.9 ± 5.74	53.91 ± 6.71	0.731	0.128
Duration of MV [d, M (P25, P75)]	6 (4,8)	15 (12,17)	−6.87	<0.001
paO_2_/FiO_2_ (mmHg, *Mean ± SD*)	226.94 ± 50	239.65 ± 32	−0.489	0.626
PH (*Mean ± SD*)	7.44 ± 0.52	7.47 ± 0.74	−1.570	0.120
bla (mmol/L, *Mean ± SD*)	1.13 ± 0.54	1.54 ± 0.23	−1.787	0.081
K^+^ (mmol/L, *Mean ± SD*)	4 ± 0.61	3.83 ± 0.71	*1.207*	0.231
Ca2 + (mmol/L, *Mean ± SD*)	1.29 ± 0.17	1.29 ± 0.19	0.180	0.858
Causes of MV (*n*, %)			*4.307*	0.366
ACD (*n*, %)	14 (27.5)	6 (17.1)		
AHF (*n*, %)	12 (23.5)	11 (31.4)		
Pneumonia (*n*, %)	12 (23.5)	12 (34.3)		
Post-operation (*n*, %)	8 (15.7)	2 (5.7)		
The other (*n*, %)	5 (9.8)	4 (11.4)		

BMI, body mass index; APACHEII, acute physiology and chronic health; SOFA, sequential organ failure assessment; HR, heart rate; RR, respiratory rate; VT, tidal volume; MV, mechanical ventilation; bla, blood lactic acid; ACD, acute cerebrovascular diseases; AHF, acute heart failure; MAP, mean arterial pressure; EF, ejection fraction.

We performed the diaphragmatic assessment by ultrasound. Table 2 summarizes the relevant data assessed by ultrasound. Compared to the successful weaning group, the weaning failure group showed significant decreases in Whole Strain (*p* < 0.001), DIcos Strain (*p* < 0.001), DIcru Strain (*p* = 0.001), DE (*p* < 0.001), and DTF (*p* < 0.001). Although DIdome Strain showed a decreasing trend, there was no statistical difference between the two groups (*p* = 0.334), and there was no statistical difference in DTei and DTee between the two groups.

**Table 2:** Results of the ultrasonographic assessment of diaphragmatic function.

Parameter	Weaning success group (*n* = 51)	Weaning failure group (*n* = 35)	*t*-values	*p*-values
Whole Strain (%)	−2.3 ± 1.12	−1.38 ± 0.91	−3.973	<0.001
DIcos Strain (%)	−12.6 ± 5.34	−7.79 ± 4.14	−4.493	<0.001
DIdome Strain (%)	10.03 ± 2.47	9.72 ± 4.13	0.971	0.334
DIcru Strain (%)	−11.08 ± 4.38	−7.54 ± 5.35	−3.35	0.001
DE (cm)	1.71 ± 0.45	1.01 ± 0.35	5.209	<0.001
DTei (cm)	0.237 ± 0.06	0.229 ± 0.06	0.556	0.580
DTee (cm)	0.176 ± 0.05	0.187 ± 0.05	−0.862	0.392
DTF (%)	37.91 ± 8.76	24.75 ± 7.12	3.027	0.001

ST, speckle tracking; DE, diaphragm excursion; DTei, end-expiratory diaphragm thickness; DTF, diaphragm thickening fraction; DTee, end-expiratory diaphragm thickness; Dlcos, costal diaphragm; DIdome, diaphragmatic dome; Dlcru, crural diaphragm.

**Figure 3:**
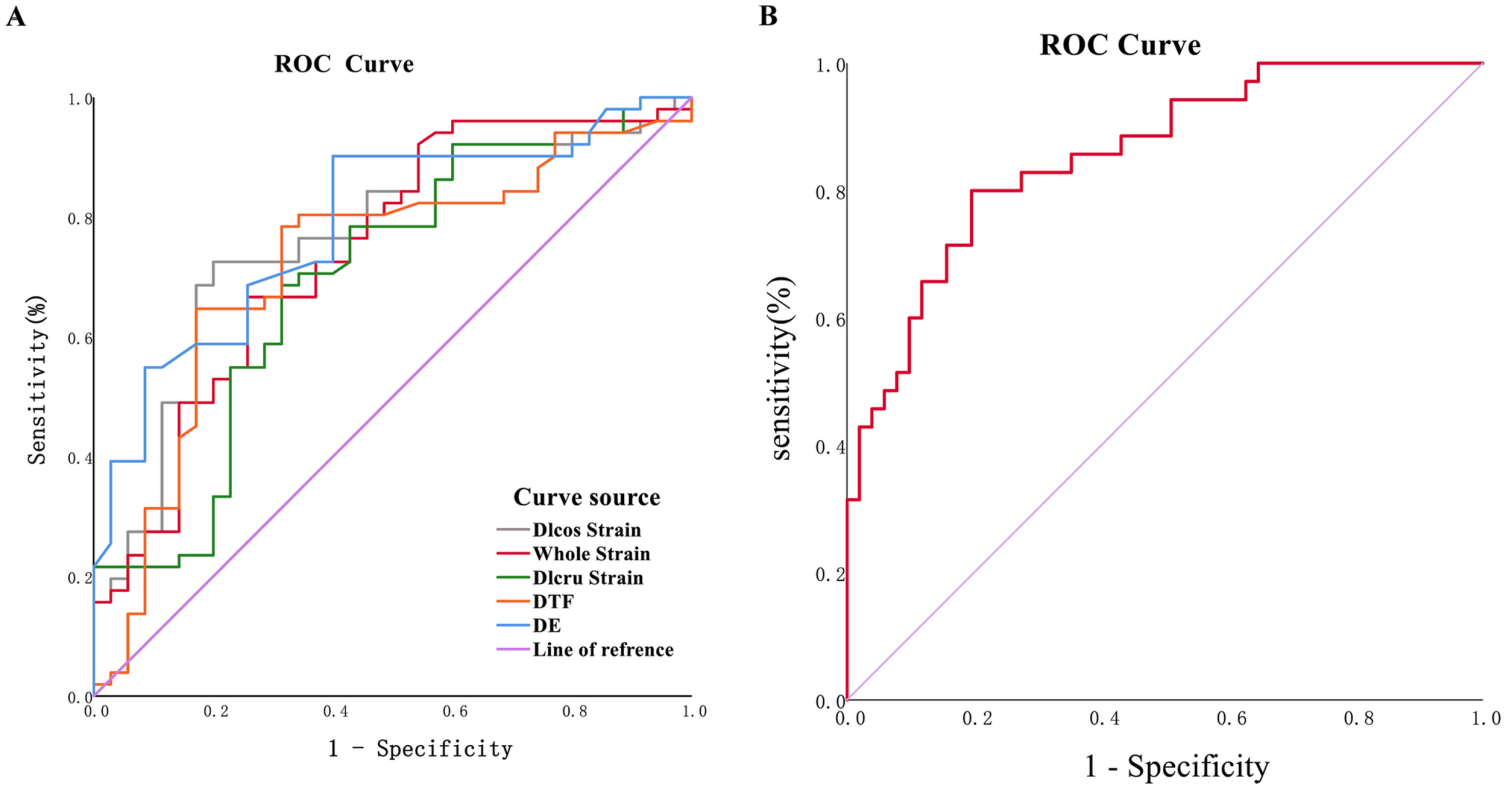
Ultrasonographic ROC curve. **(A)** ROC curve of single-indicator; **(B)** ROC curve of DE joint whole strain.

To further evaluate the predictive value of these variables for weaning outcomes, ROC analysis was conducted. The results showed that a Dlcos strain value exceeding −9.836 had a higher diagnostic value (Figure 3A; Table 3), with an area under the curve (AUC) of 0.760, and a sensitivity and specificity for predicting successful weaning of 80 and 72.5%, respectively. A DE value exceeding 1.015 cm had an AUC of 0.785, indicating that DE value had a high specificity (90.2%) for predicting successful weaning, but a lower sensitivity (60%) (Figure 3; Table 3). Furthermore, DE was combined with Whole Strain, the AUC value was 0.856, and the sensitivity (80%) and specificity (80.4%) were more balanced compared to using DE alone (Figure 3B).

**Table 3:** Predictive value of strain, strain rate and displacement on weaning failure.

Test variable	Area	*p*-value	95% Confidence interval	Sensitivity (%)	Specificity (%)	Cut-off
DIcos strain	0.760	<0.001	0.656–0.865	80	72.5	−9.835
Whole strain	0.740	<0.001	0.631–0.868	74.3	66.7	−1.74
DIcru strain	0.698	0.002	0.583–0.813	68.6	68.6	−9.026
DE	0.785	<0.001	0.689–0.882	90.2	60	1.015
DTF	0.721	0.001	0.607–0.835	82.9	64.7	29

Next, we performed univariate regression analyses. The results showed that Whole strain, Dlcos strain, Dlcru strain, DE and DTF were strongly associated with weaning outcomes. Furthermore, we performed multivariate logistic regression analysis to identify independent predictors of weaning outcomes. We found that whole strain (odds ratio [OR]: 1.962, 95% confidence interval [CI]: 1.042–3.655, *p* = 0.037) and DE (OR: 0.107, 95% CI: 0.024–0.486, *p* = 0.004) were independent predictors of weaning outcomes in ICU patients. The results of univariate regression analyses and the logistic regression analysis are shown in Table 4.

**Table 4:** Univariate and multivariate analyses comparing weaning indicators predicting weaning outcomes.

Variable	OR, 95%CI	*p*	OR, 95%CI	*p*
Whole strain	2.488 (1.475–4.196)	0.001	2.555 (1.476–4.425)	0.001
Dlcos strain	1.236 (1.106–1.381)	<0.001	–	–
Dlcru strain	1.195 (1.016–1.347)	0.003	–	–
DE	0.112 (0.035–0.360)	<0.001	0.084 (0.020–0.347)	0.001
DTF	0.961 (0.934–0.989)	0.006	–	–

## Discussion

Accurately determining the optimal timing for weaning in the critical care setting presents a significant challenge. SBT is an important method to guide the liberation from mechanical ventilation for ICU patient (15). However, the limitations of SBT restrict its accuracy in predicting weaning outcomes, leading to prolonged mechanical ventilation or premature extubation, and even reintubation. The limitations of SBT include, firstly, heavy reliance on subjective judgment from physicians and the subjective experience of patients (16); secondly, although some objective criteria are established in SBT, these indicators may lack specificity. For instance, an increased heart rate is an important indicator in SBT evaluation and a risk factor for reintubation, but the elevation in heart rate could be attributable to stress rather than intolerance to hypoxia (17). Furthermore, impaired diaphragm function could be a critical factor in SBT failure (18, 19). Therefore, this study combines speckle tracking technology with traditional ultrasound to evaluate diaphragm function and attempts to guide the extubation process in patients.

In mechanically ventilated patients, diaphragmatic dysfunction can lead to inadequate ventilation, decreased lung capacity, and prolonged mechanical ventilation. Traditional ultrasound assessments rely on parameters such as DE and DTF to evaluate diaphragmatic function. However, recent studies have shown that DE itself is not a reliable indicator of diaphragmatic contraction strength. On one hand, diaphragmatic displacement is significantly influenced by inspiratory volume (7, 20). On the other hand, factors such as impedance of neighboring structures and abdominal compliance can affect diaphragmatic motion during inspiration (21). DTF is a more challenging measurement parameter because the diaphragm is thin and easily influenced by minimal changes in measurement, which can severely impact the results (22). Interestingly, a multicenter study showed that DE and DTF diaphragmatic ultrasound could not predict extubation failure (23). This may be due to traditional ultrasound assessment parameters are static and unable to capture the contraction motion characteristics of the diaphragm throughout the respiratory cycle. Nonetheless, in this study we found that DE value exceeding 1.015 cm had an AUC of 0.785, indicating that DE value had a high specificity (90.2%) for predicting successful weaning, but a lower sensitivity (60%). Our result is similar to the result of a previous meta-analysis (24).

Speckle tracking ultrasound technology provides a promising solution. This technology involves frame-by-frame tracking of spatial motion of muscle fiber tissue and reveals tissue motion by analyzing the spatial motion trajectory of specific points on ultrasound images (25). It can detect and track the deformation of fiber tissue over time, thereby providing a more accurate evaluation of diaphragmatic function (16). Preliminary studies have also confirmed that speckle tracked diaphragmatic strain quantification is readily available in both healthy subjects and mechanically ventilated patients (26, 27). In summary, this technology offers a more precise evaluation of diaphragmatic function by tracking the motion of points within ultrasound images throughout the entire respiratory cycle.

The muscular portion of the diaphragm includes the costal diaphragm (Dlcos) and the crural diaphragm (Dlcru), which exhibit apparent differences in development, anatomical structure, and function. Previous studies have shown that the strain indicator of active diaphragmatic contraction initially occurs at the diaphragmatic rib edge and subsequently at the diaphragmatic crus during inspiration (28). These differences can be attributed to variations in diaphragm thickness, fiber length, and region of rib edge shortening (29). In this study, we aim to assess diaphragmatic function in mechanically ventilated patients and predict weaning outcomes using speckle tracking ultrasound technology. Our study results show that a Dlcos strain value exceeding −9.836 has good predictive value, with significantly improved sensitivity compared to DE (80% vs. 60%), albeit slightly lower specificity (72.5% vs. 90.2%). This indicates that Dlcos strain aids in identifying patients at risk of weaning failure. The prediction performance of the DIcru strain is less than Dlcos strain. In addition to the tracking of the Dlcos strain and the Dlcru strain, the speckle tracking ultrasound can also complete the tracking of the DIdome strain. Unfortunately, in the results of our study, DIdome strain failed to predict weaning outcomes, suggesting that DIdome strain may not be suitable for evaluating diaphragmatic function. Furthermore, combining speckle tracking with traditional ultrasound reveals a better predictive value when DE is combined with Whole Strain (AUC: 0.856), with enhanced balance between sensitivity and specificity. This demonstrates the application value of combining speckle tracking ultrasound with traditional ultrasound. It should be noted that implementing speckle tracking ultrasound evaluation faces numerous challenges. One important challenge is ensuring the accuracy and consistency of measurements through standardized measurement techniques, which are crucial for clinical use. Additionally, personnel require extensive specialized training. Furthermore, further research is needed to determine the populations that can benefit from speckle tracking and applicable clinical scenarios.

There are limitations to the present study. Firstly, this study is a single-center study. The single-center design and small sample size may introduce biases in the research results, emphasizing the need for larger and multicenter studies. Secondly, our study only measured the right hemidiaphragm due to its relative ease and accuracy of measurement compared to the left hemidiaphragm, which limits the applicability of this study in patients with unilateral diaphragmatic paralysis. Thirdly, the speckle tracking algorithm was initially designed for analyzing myocardial tissue. When observing the diaphragm, readjusting or defining the regions of interest (ROI) may be necessary to adequately track the diaphragm. This adjustment could introduce corresponding errors, highlighting the need for algorithm optimization. Finally, Due to the invasive nature of the procedure, the complexity of the procedure, some risk of infection, and some individual differences, our study was the lack of pdi data (tarns-diaphragmatic pressure).

In summary, the application of Speckle tracking ultrasound for the assessment of diaphragm function represents a novel approach in predicting weaning readiness among critically ill patients. Unlike traditional methods, this technique offers objective and dynamic measurement data, unaffected by subjectivity or inter-operator variability. The potential of Speckle tracking ultrasound lies in its ability to enhance weaning decisions and ultimately improve patient outcomes. Nevertheless, to establish its value in routine clinical practice, further research and standardization are imperative.

## Conclusion

Speckle tracking ultrasound has emerged as a novel tool for assessing cardiac function. In this study, we employed this technique to evaluate diaphragmatic function among mechanically ventilated patients. The findings of this study demonstrate the feasibility of using speckle tracking ultrasound to assess diaphragmatic function in mechanically ventilated patients. The combined utilization of whole strain and DE provides a more precise evaluation of diaphragmatic function in ICU patients, which may improve patient outcomes.

## Data Availability

The original contributions presented in the study are included in the article/supplementary material, further inquiries can be directed to the corresponding authors.
